# Novel *CUL7* biallelic mutations alter the skeletal phenotype of 3M syndrome

**DOI:** 10.1038/s41439-020-0090-6

**Published:** 2020-02-04

**Authors:** Nao Takizaki, Yoshinori Tsurusaki, Kaoru Katsumata, Yumi Enomoto, Hiroaki Murakami, Koji Muroya, Hiroshi Ishikawa, Noriko Aida, Gen Nishimura, Kenji Kurosawa

**Affiliations:** 1 Division of Medical Genetics, Kanagawa Children’s Medical Center, Yokohama, Japan; 2 Clinical Research Institute, Kanagawa Children’s Medical Center, Yokohama, Japan; 3 Department of Neonatology, Kanagawa Children’s Medical Center, Yokohama, Japan; 4 Department of Endocrinology and Metabolism, Kanagawa Children’s Medical Center, Yokohama, Japan; 5 Department of Obstetrics and Gynecology, Kanagawa Children’s Medical Center, Yokohama, Japan; 6 Intractable Disease Center, Saitama Medical University Hospital, Saitama, Japan; 7 Department of Radiology, Kanagawa Children’s Medical Center, Yokohama, Japan

**Keywords:** Growth disorders, Bone

## Abstract

3M syndrome is an autosomal recessive disorder characterized by severe growth retardation, distinct facial features, and skeletal changes, including long slender tubular bones and tall vertebral bodies. We report a Japanese patient with 3M syndrome caused by the biallelic novel variants c.1705_1708del and c.1989_1999del of *CUL7*. Skeletal features were consistent with 3M syndrome in the early neonatal period but became less obvious by 2 years of age.

3M syndrome (MIM #273750) is a rare autosomal recessive disorder characterized by severe growth retardation, distinct facial features, and skeletal changes. Skeletal manifestations include long slender tubular bones and tall vertebral bodies^1^. Causative germline biallelic mutations have been identified in the cullin 7 (*CUL7*) (MIM #273750)^2^, obscurin like 1 (*OBSL1*) (MIM #612921)^3^, and coiled-coil domain containing 8 (*CCDC8*) (MIM #614205)^4^ genes. *CUL7* encodes a component of an E3 ubiquitin-protein ligase complex. In brief, CUL7 interacts with the tumor suppressor protein p53, cullin 9 (CUL9), and F-box and WD repeat domain containing 8 (FBXW8) proteins, leading to the regulation of microtubules and genome stability^5,6^.

Here, we report a patient with novel compound heterozygous mutations who developed less-severe distinct skeletal features during the neonatal period until 2 years of age.

The proband was a 1-year-old Japanese boy born to nonconsanguineous parents with no family history of bone dysplasia. Fetal ultrasonography showed a severely short femur of 21 mm (−2.6 SD) at 19 weeks. Progressive fetal growth retardation suggested skeletal dysplasia at 21 weeks of gestation. At 28 weeks, severe shortening of the femur with −5 SD, relative macrocephaly, and short thorax were noted (Fig. 1a).

**Fig. 1:**
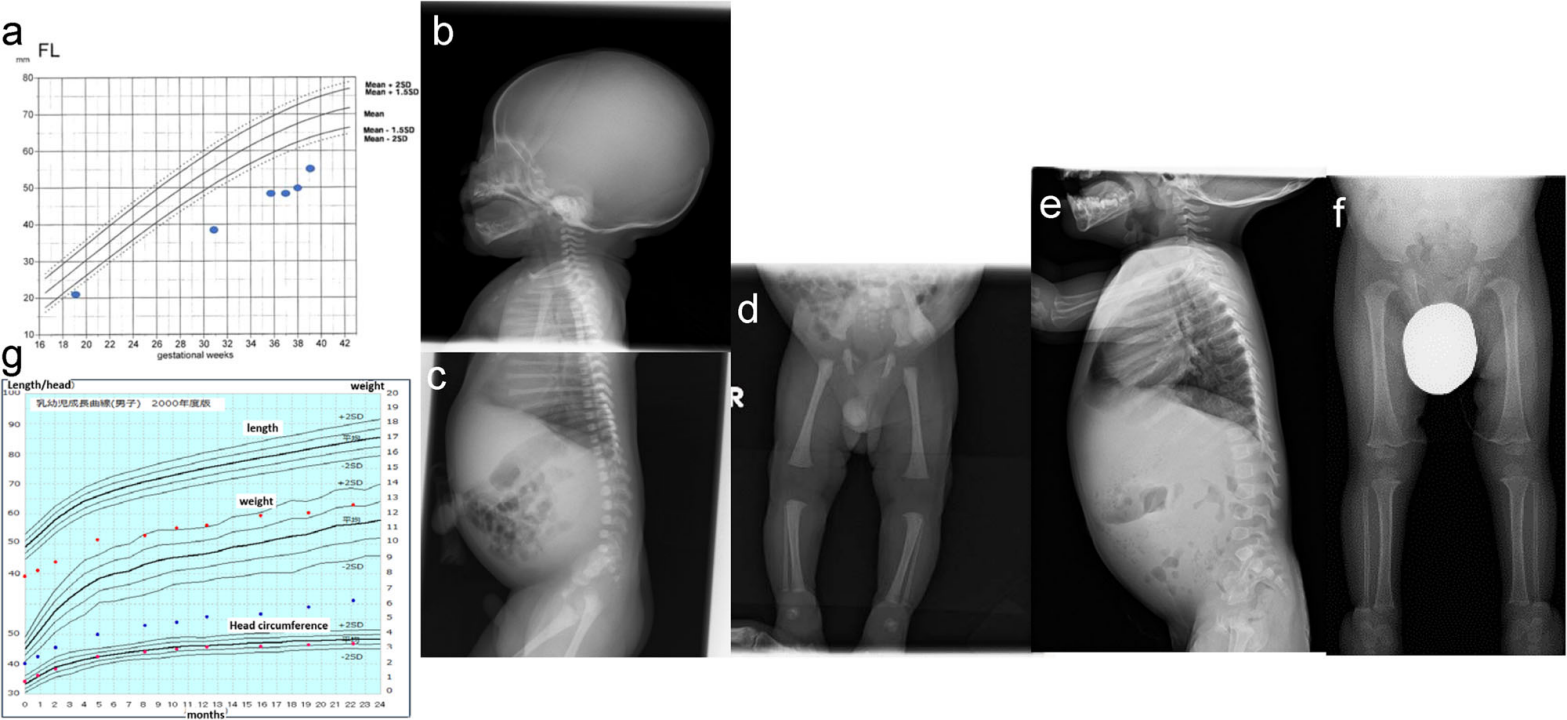
Pre- and postnatal growth patterns and skeletal surveys. **a** Growth pattern of fetal femur lengths. Compared with the standard, differences in femur length increased with gestational age. **b**–**d** Skeletal survey at birth. Tall vertebral bodies and slender tubular bones were noted. **e**–**f** At 1 year and 3 months of age, skeletal features, such as tubular bones and increased height of vertebral bodies, were not as prominent. **g** Postnatal growth curve by 2 years of age. The growth pattern showed severe short stature but standard head circumference.

He was born at 39 weeks of gestation by emergency cesarean section because of his non-reassuring fetal status. His birth weight was 2007 g (−3.5 SD), length was 39.0 cm (−4.8 SD), occipital frontal circumference was 34 cm (+0.6 SD), and chest circumference was 26 cm. Apgar scores were 8/9. Physical examination at birth revealed a phenotype of relative macrocephaly, triangular face with low nasal root, short neck, and short limbs with prominent heels. Concomitantly, a bone survey at birth revealed tall vertebral bodies and slender long bones (Fig. 1b–d). He spent 3 days in the neonatal intensive care unit with no medical support and was discharged to his mother on day 8. Notably, at 1 year and 3 months, reevaluation of skeletal X-rays showed mild manifestations for typical 3M syndrome compared with those observed at birth (Fig. 1e, f). At the age of 1 year and 10 months, his weight was 6180 g (−4.3 SD), length was 62.8 cm (−7.3 SD), occipital frontal circumference was 46.5 cm (−1.1 SD), and chest circumference was 31.4 cm (Fig. 1g). According to the expected developmental milestones, the patient demonstrated mild developmental delay for his age, with eye contact at 4 months, rolling over at 8 months, sitting without support for 10 months, and speaking meaningful words and walking without support at 15 months.

Clinical information was collected after obtaining written informed consent from the patient’s family. This study was approved by the institutional review board of Kanagawa Children’s Medical Center. Genomic DNA was extracted from the peripheral blood of the patient and both parents using the QIAcube Kit (QIAGEN, Hilden, Germany) according to the manufacturer’s instructions. Targeted resequencing was performed for the affected patient. Genomic DNA captured by the TruSight One Sequencing Panel (Illumina, San Diego, CA, USA) was sequenced on the MiSeq platform (Illumina) with 151-base pair paired-end reads as previously described^7^. Candidate variants were confirmed by Sanger sequencing.

We identified the compound heterozygous variants NM_014780.4:c.1705_1708del:p.(Gly569Leufs*4) and c.1989_1999del:p.(Gln664Glyfs*12) of *CUL7*. Sanger sequencing confirmed that the c.1705_1708del variant was inherited from the father, whereas the c.1989_1999del variant was inherited from the mother (Fig. 2). These variants were novel and are not included in the public databases gnomAD, Tommo, ClinVar, Human Genetic Variation Database, and Human Genome Mutation Database 2019.4. Both identified *CUL7* variants were predicted to result in a premature termination codon consistent with biallelic loss-of-function mutations.

**Fig. 2:**
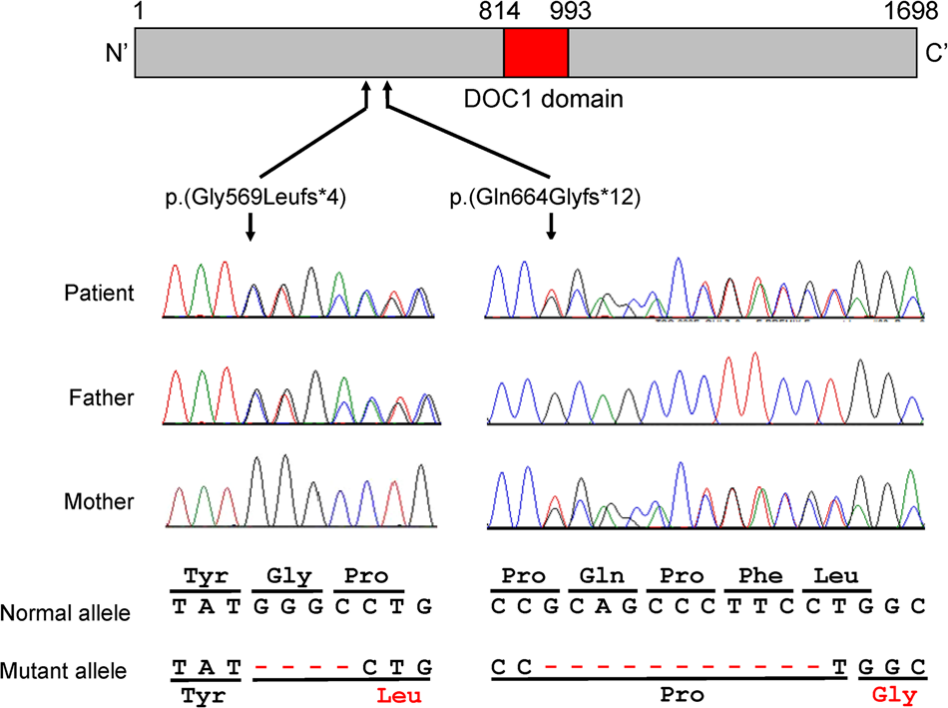
Schematic representation of CUL7 protein and compound heterozygous variants in *CUL7*. CUL7 contains the DOC1 domain, interacting with various ligands. Electropherograms revealed biallelic variants c.1705_1708del:p.(Gly569Leufs*4) and c.1989_1999del:p.(Gln664Glyfs*12) derived from the father and mother, respectively.

The prenatal growth patterns of 3M syndrome are associated with severe limb shortening^8^. The skeletal features have been suggested to become less-distinct with age^2^. However, little is known about the age and process of these skeletal changes. Although the proband exhibited the cardinal skeletal features of 3M syndrome during both the prenatal and neonatal periods, his skeletal features of long tubular bones and tall vertebral bodies were less apparent by 2 years of age. This indicates that normalization of skeletal features, particularly the shape of vertebral bodies, can occur over 1 year of age.

There are generally no genotype-to-phenotype correlations in patients with 3M syndrome^9^. Skeletal changes and growth patterns are variable, even in the same family^10^. In agreement with these reports, our results suggest that the skeletal features of 3M syndrome vary depending on the patient’s developmental age and motor functions.

CUL7 consists of 1698 amino acids. CUL7 has a DOC1 domain and C-terminated Cullin domain associated with FBXW8 binding and ROC1 binding, contributing to mitosis and cytokinesis^6^. Aberrant transcripts of truncating *CUL7* variants are expressed in the fibroblasts of patients^2^. Although we could not assess the transcripts from both abnormal alleles, the biallelic loss-of-function mutations of *CUL7* in our patient were believed to perturb mitosis and cytokinesis, resulting in the 3M syndrome phenotype.

In conclusion, we identified novel truncating mutations of *CUL7* in a Japanese patient with 3M syndrome. Skeletal surveys revealed changing manifestations with progressively less-distinct features of the long bones and vertebrae from the neonatal to early infantile period by 2 years of age. To understand the mechanisms of these changes and observed variability in the growth patterns during the pre- and postnatal periods with or without growth hormone treatment^11^, further studies combined with skeletal surveys and growth curve analysis of patients with 3M syndrome are required.

## Data Availability

The relevant data from this Data Report are hosted at the Human Genome Variation Database at 10.6084/m9.figshare.hgv.2805, 10.6084/m9.figshare.hgv.2808.
